# Manipulation of visible-light polarization with dendritic cell-cluster metasurfaces

**DOI:** 10.1038/s41598-018-28030-8

**Published:** 2018-06-26

**Authors:** Zhen-Hua Fang, Huan Chen, Di An, Chun-Rong Luo, Xiao-Peng Zhao

**Affiliations:** Smart Materials Laboratory, Department of Applied Physics, Northwestern Polytechnical University, Xi’an, 710129 P. R. China

## Abstract

Cross-polarization conversion plays an important role in visible light manipulation. Metasurface with asymmetric structure can be used to achieve polarization conversion of linearly polarized light. Based on this, we design a quasi-periodic dendritic metasurface model composed of asymmetric dendritic cells. The simulation indicates that the asymmetric dendritic structure can vertically rotate the polarization direction of the linear polarization wave in visible light. Silver dendritic cell-cluster metasurface samples were prepared by the bottom-up electrochemical deposition. It experimentally proved that they could realize the cross - polarization conversion in visible light. Cross-polarized propagating light is deflected into anomalous refraction channels. Dendritic cell-cluster metasurface with asymmetric quasi-periodic structure conveys significance in cross-polarization conversion research and features extensive practical application prospect and development potential.

## Introduction

Manipulating light polarization is always desirable in practical applications. Visible light will possibly become the main medium of communication and information processing in the next generation^1,2^. In recent years, researchers attempted to manipulate light through various means. Artificial design of different cell structures of metamaterials allowed them to acquire many characteristics that are nonexistent in nature; such characteristics include negative refraction, anomalous Cerenkov radiation, anomalous Doppler effect, perfect lens, super-resolution imaging, invisibility cloaking, and electromagnetic-wave polarization rotation^3–11^. These characteristics attract more and more researchers to study metamaterials, particularly those operating at microwave, infrared^12,13^, and visible light^14^ wavelengths. As two-dimensional metamaterials, metasurfaces preserve characteristics of their three-dimensional counterparts in manipulating electromagnetic-wave behavior while reducing challenges in fabrication^15^. Ultrathin metasurfaces can now be easily designed to deflect a propagating light into anomalous refraction channels^16–23^, thereby obeying generalized Snell’s law by imparting phase discontinuities. Metasurface thickness is much smaller than operational wavelength, theoretically allowing miniaturization and integration of optical components^24^. Lee *et al*.^25^ proposed and fabricated metasurfaces based on coupling of electromagnetic modes in plasmonic metasurfaces with quantum-engineered electronic intersubband transitions in semiconductor heterostructures.

Polarization is an important characteristic of light, and recent efforts were exerted to control light polarization through arrays of nanoantennas, plasmonics, and dielectrics^26–35^. Lin *et al*.^26^ reported that dielectric gradient metasurface optical elements can also achieve high efficiencies in transmission mode in the visible spectrum. Considerable progress was attained in cross-polarization rotation within the frequency range of visible light. Qin *et al*.^24^ revealed that cross-polarization conversion efficiency can be increased to 36.5% by optimizing the proposed Hybrid bilayer plasmonic metasurface structure at 815 nm wavelength. It is admirable that the top-down metasurfaces behave 99% polarization control efficiency, 99% phase control efficiency and over 90% total energy efficiency in infrared^30,31^. Other studies on cross-polarization conversion in short-wavelength visible light revealed significant development potential. For example, Gansel^32^ investigated light propagation through a uniaxial photonic metamaterial comprising three-dimensional gold helices arranged on a two-dimensional square lattice. These nanostructures were fabricated using direct laser writing into a positive-tone photoresist followed by electrochemical deposition of gold. However, a majority of the metasurfaces has been published are prepared by top-down mechanical etching methods^33–35^, such as ion beam lithography, photolithography and photoetching. The expensive equipment, harsh experimental conditions, complex preparation process and the restricted sample size limiting the practical application of the metasurface with nanoscale fine structure in the visible light. For the electrochemical preparation method, equipment and preparation process is simple, low cost and can be easily used for large area preparation^36,37^. The silver dendritic metasurface samples were prepared by this method with a unified shape of dendritic structure, and the resonant wavelengths were adjustable in the visible light^38^.

In the present work, bottom-up electrochemical deposition is used to prepare a dendritic cell-cluster metasurface that can achieve effective cross-polarization conversion in transmission mode. The method does not require expensive equipment or harsh conditions. Significant cross-polarization conversion is achieved at visible-light wavelengths of 550, 570, 590, and 620 nm. Cross-polarized transmitted light is deflected from normal when it passes through the dendritic cell-cluster metasurface. These results demonstrate significant improvement in visible-light manipulation.

## Experimental details

**Figure 1:**
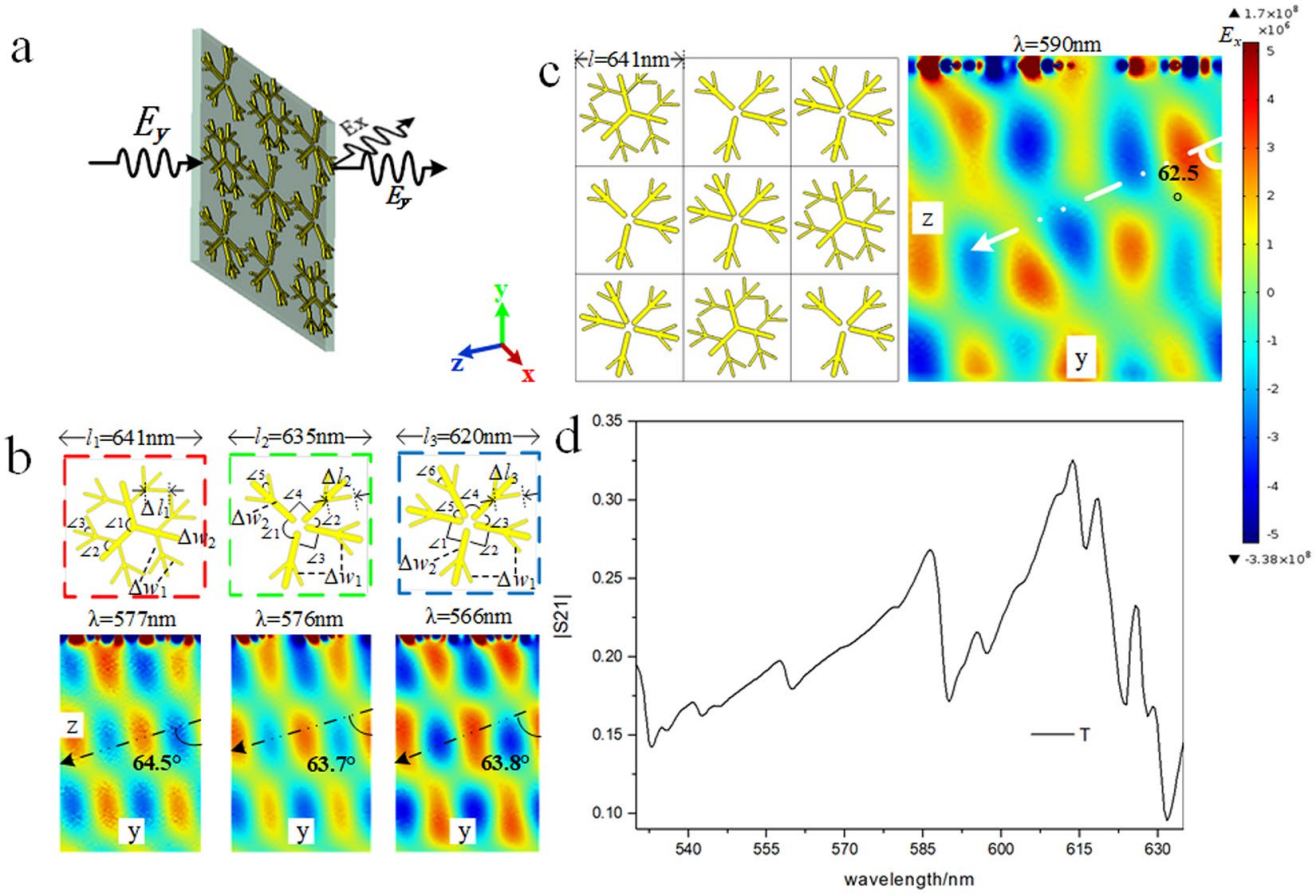
Dendritic structure and numerical simulation. (**a**) Schematic of a linearly polarized plane wave perpendicularly passing through the dendritic cell-cluster metasurface. (**b**) Schematic of three types of dendritic cells and their transmitted electric field distribution. (**c**) Schematic of a dendritic cell-cluster comprising three types of dendritic cells. Transmitted electric field of dendritic cell-cluster metasurface; (**d**) Transmitted spectrum of dendritic cell-cluster metasurface.

**Figure 2:**
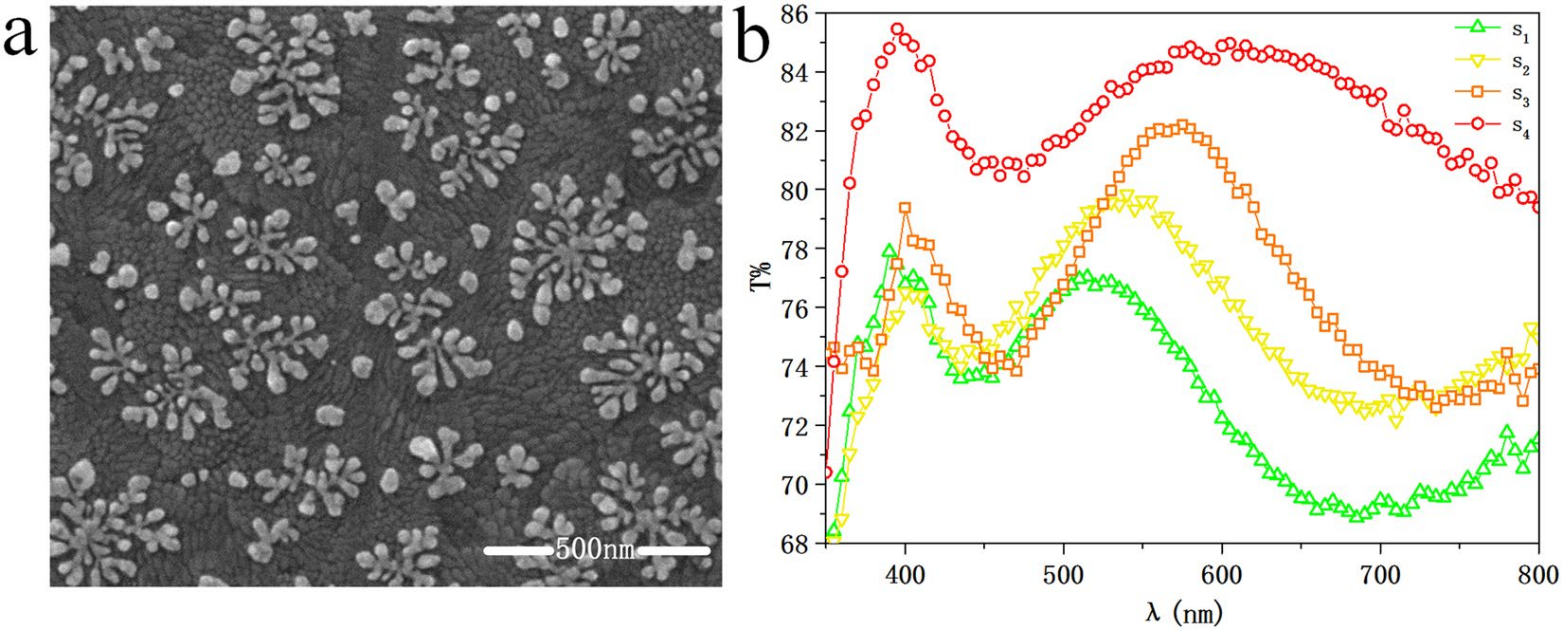
Dendritic metasurface sample characterization analysis. (**a**) SEM photograph of partial silver dendritic cell-cluster metasurface. The scale label is 500 nm. (**b**) The transmission spectrum of four dendritic cell-cluster metasurface samples.

COMSOL Multiphysics is based on the finite element method, and it has been widely used in many fields to solve the physical phenomenon of the real world by mathematical method. COMSOL Multiphysics is used to simulate the transmission of dendritic cell-cluster metasurface in this paper. The material on SiO_2_ substrate is dendritic Ag, whose relative permittivity can be described by the Drude model with a plasma frequency of *ω*_*pl*_ = 1.37 × 10^16^ s^−1^ and a collision frequency of *ω*_*col*_ = 8.5 × 10^13^ s^−1^. Thicknesses of dendritic structure is 12 nm. We simulated and calculated lots of metasurface models which the thickness of the substrate increases from tens of nanometers to several micrometers. The simulation results are nearly identical. It can be seen that the thickness of the substrate has almost no influence on the optical response behavior of the metasurface, so the substrate thickness of the actual sample is applying to a wide range. Periodic boundary conditions were used in the x- and y-axes, and an open boundary condition was used in the z-axis. Incident wave in all cases is set as a linearly polarized plane wave ***E***_***y***_ perpendicularly transmitted to the surface along −*z*, as shown in Fig. 1. The preparation experiments were carried out with an electrochemical workstation. Dendritic Ag layer was grown on indium tin oxide (ITO). The constant deposition voltage is 0.9 V. The electrolyte is a mixed solution of AgNO3 (0.1 mg/mL) and polyethylene glycol-20000 (PEG-20000, 0.12 g/mL). Metasurface samples responding to different wavelengths of visible light are obtained by adjusting deposition time. The resonant wavelength of dendritic metasurface is measured in the spectrophotometer. Scanning electron micrograph of the dendritic structure is zoomed in at a magnification of 2 × 10^5^. The meta-atom in the real metasurface sample is randomly distributed, and the number of the units in is 10^9^/cm^2^. After repeated experiment preparation and transmission spectrum test, when the obvious resonant wavelength appears, a large number of units in the sample are quasi-periodic distribution, as shown in Fig. 2a. Therefore, periodic boundary conditions can be used in the simulation process.

**Figure 3:**
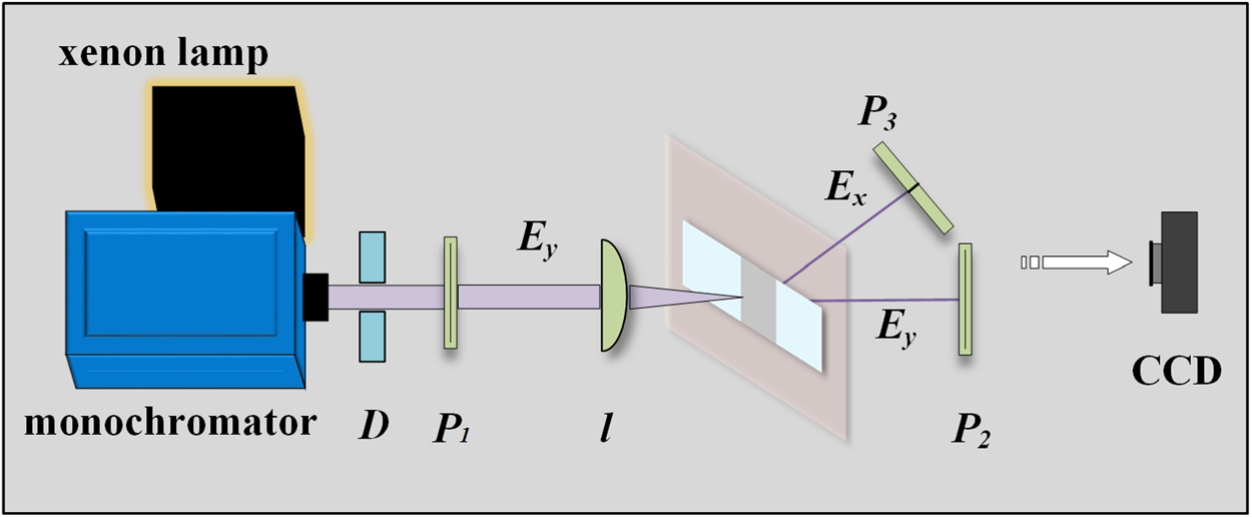
Experimental setup used to measure transmitted light through the metasurface.

Optical transmission of the dendritic cell-cluster metasurface is measured using a tunable broadband source. Figure 3 shows the schematic of experimental setup. A xenon light coupled with a visible-near-infrared monochromator serves as the tunable light source (wavelength range = 300–2000 nm). The tunable light source was used in the experiment to ensure that the sample had an anomalous effect on incident light with a wavelength consistent with the resonant band when the experimental conditions were determined and that there was no anomalous effect in the non-resonant band. The wavelength of incident light varies from 490 nm to 640 nm with a step length of 10 nm. The plane wave generated from the monochromator is circularly polarized. Then the light from the monochromator passed through a polarizer P1, so the light entering the sample was linearly polarized light ***E***_***y***_, which was set in the comsol simulation process. The sample of dendritic cell-cluster metasurface to be measured is placed perpendicular to the incident light. The linearly polarized incident light ***E***_***y***_ is focused on the surface of dendritic cell-cluster metasurface using a planoconvex lens (focal length = 50 mm). Focal spot size is approximately 2 mm, probably covering more than 10^8^ individual dendritic elements. Because the weak interaction of units can be ignored, the certain anomalous effect will present via automatic contrast selection of the statistical effects. Polarizers P2 and P3 behind the sample are used to detect the polarized direction of transmitted light, and transmitted light spot is received by a thin semitransparent film. All elements of the experiment are placed on a self-balancing optical table, and measurement is performed in an optical darkroom.

## Results and Discussion

Theory and experiment have proved that dendritic structure is actually a combination of rod and split ring, negative ε and negative μ can simultaneously be achieved in microwave or infrared^13,36^. And with the concept of metasurface proposed in Science^15^, the authors use eight basic elements of the rod and V-shaped to achieve generalized Snell’s law. Relevant experiments and theories^12,39,40^ show that the metamaterial is a weak interaction system, in which the interaction between the structural units is weak. And thus the interaction of the rod and V-shaped split ring structure in the dendritic metasurface is negligible. The typical method derived from the reference^15^ is used to simulate the dendritic metasurface. The obtained electric field distribution has a statistical effect. The model of dendritic cell-cluster has discussed in the appendix and our previous paper^38^. The results show that the propagation of light through the dendritic metasurface coincides with the generalized Snell’s law, the interrelation between the refraction angle and the incident angle is different from the classical refraction law.

Figure 1a shows the samples horizontally placed in the *XY* plane. Periodic boundary conditions are used in *x* and *y* directions, and an open boundary condition is used in the *z*-direction. Figure 1b shows detailed structures of the three types of dendritic metasurfaces and electric field in the *x*-direction of the transmitted wave. The three types of dendritic cells are asymmetric. The dotted arrows indicate the direction of the transmitted wave. For more detailed geometric parameters of dendritic structure please refer the appendix. Wavelengths of light for the three types of dendritic structures are 577, 576, and 566 nm, respectively. The length of units is *l*_1_ = 641 nm, *l*_2_ = 635 nm, *l*_3_ = 620 nm. A linearly polarized transmitted plane wave ***E***_***x***_ is obtained and deflected into the anomalous refraction channel. The refraction angles are 64.5°, 63.7° and 63.8°, respectively. The wavelengths of incident light and the refraction angles of the three types dendritic structure are depending on the size, morphology and structure density of the dendritic units. For simulation of the actual sample structure, the three types of dendritic structures are randomly arranged to form a dendritic cell-cluster, and the cluster contains three of each type of dendritic structure (Fig. 1c). The side length of the structural element has an important influence on the resonant wavelength of the sample. The length of different types of dendritic cells are set as *l* = 641 nm in the dendritic cell cluster which resulting in an operating wavelength of 590 nm. Refer to the real sample, the size, fine structure and distribution density of the dendritic cell are non-uniform in addition to the unified shape of dendritic structure. Figure 1d displays the transmitted electric field of the dendritic cell-cluster metasurface. A cross-polarized transmitted plane wave ***E***_***x***_ is also obtained, and the refraction angle is 62.5°. After determining the morphology, size, and distribution of the dendritic structure, the anomalous refraction angle is determined. From the above results, it can be seen that the refraction angle can be controlled by changing the topography, size and distribution density of the dendritic structure. The distribution density of the dendritic units determines the angle of the anomalous refraction. When the three types of dendritic structures were combined, the length of different types of dendritic cells are set as unified value *l* = 641 nm in the dendritic cell cluster, *l* = *l*_1_ = 641 nm, *l* > *l*_2_ > *l*_3_, the refraction angle became smaller along with the density of the dendritic units decreased. The operation wavelength of the dendritic cell cluster measures 590 nm. More resonant wavelength can also be realized by adjusting the model size and so on. Simulation results suggest that dendritic cell-cluster metasurface can manipulate light by cross-polarized conversion and negative refraction.

The dendritic cell-cluster metasurface sample consists of three layers: a bottom layer, which is a substrate made of indium tin oxide (ITO) conductive glass; an interface layer, which is composed of evenly distributed individual 2D silver dendritic cells (Fig. 2a), the structure of dendritic metasurface is asymmetric; and a top layer, which is an oxidation-resistant coating formed by Polyvinyl Alcohol (PVA). ITO conductive layer is part of the substrate, PVA is the oxidation protection layer. Both the calibration element and PVA has a negligible impact on the dendritic metasurface. So they are not including in simulation to simplify the calculation model under the premise of a truthful simulation of the dendritic metasurface. Scanning electron micrograph of the dendritic structure is shown in Fig. 2a. A single dendritic unit features a diameter measuring 200–300 nm, and all units are uniformly distributed onto the substrate surface. Several units with diverse sizes and branches are coupled with one another that form clusters on the dendritic metasurface. For convenience of experimental measurement, overall dimensions of the dendritic cell-cluster metasurface measure 1 × 1.3 cm^2^. SEM test results at different locations on the sample surface indicate that the dendritic units have the same appearance at all locations, and the transmission spectra at the corresponding points are basically the same. The reproducibility of the dendritic structure in the entire sample surface is very good. In addition, the samples that were prepared repeatedly also showed that the preparation process of the dendritic metasurface is stable and can be repeatedly prepared. Transmitted spectra of the four types of dendritic cell-cluster metasurfaces (s1, s2, s3, and s4) with different resonance wavelength are shown in Fig. 2b. In addition to intrinsic transmission peak for silver at 400 nm, high transmission peaks are also found in wavelength ranges of 510–530 (s1), 530–555 (s2), 555–580 (s3), and 600–630 nm (s4). The transmitted light of the sample is the main part, and the proportion of reflected light is small, which was not considered in this experiment. Samples operating in different visible wavelength are obtained by properly increasing deposition time.

Wavelength of incident light is increased from 490 nm to 640 nm with uniform speed. All transmitted light phenomena are observed and recorded in real time with a charge-coupled device camera(CCD). At the experimental design stage, we used electro-optical sensor and CCD to make comparisons. We found that the two results are basically the same. The electro-optical sensor is convenient to measure in fixed position and orientation. However, in this experiment, the positional deviation and orientation of the photodetector are more difficult to accurately control, which has a greater impact on the experimental results. The CCD is used to measure transmitted light, which can capture all useful information for analysis and calculation. Therefore, we finally adopted the CCD measurement. Consequently, an obtained video shows the transmitted light passing through the dendritic cell-cluster metasurface with varied incident wavelengths. As shown in the obtained video of s4 (Visualization 1), two optical spots are observed on the thin semitransparent film when wavelength of incident light is within the range of resonant wavelength (600–630 nm). The spot with high brightness at the center of the thin film reveals that the normal transmitted light is perpendicular to sample interface; the one with low brightness represents anomalous transmitted light. The dendritic structure is asymmetric. As shown in the simulation results, the direction of the anomalous refraction is on the right side, and only one light spot is observed on the right side of the central maximum. Polarization analyzers P_2_ and P_3_ are used to measure polarization angles of normal and anomalous transmitted lights. Results show that polarization angle of normal transmitted light is the same as that of incident light. Polarization direction of anomalous transmitted light is perpendicular to the incident light, that is, normal transmitted light is co-polarized, and anomalous transmitted light is cross-polarized. These two optical spots reveal that the dendritic cell-cluster metasurface can deflect propagating light into anomalous refraction channels. Incident light is linearly polarized, and the dendritic structure operates in the direction of polarized incident light. Thus, only one cross-polarized spot emerges. At non-resonant wavelengths (490–590 nm), only a single light spot is observed at the center of the white plate. Measurement result of polarization analyzer reveals that the transmitted light is a co-polarized light at this wavelength.

**Figure 4:**
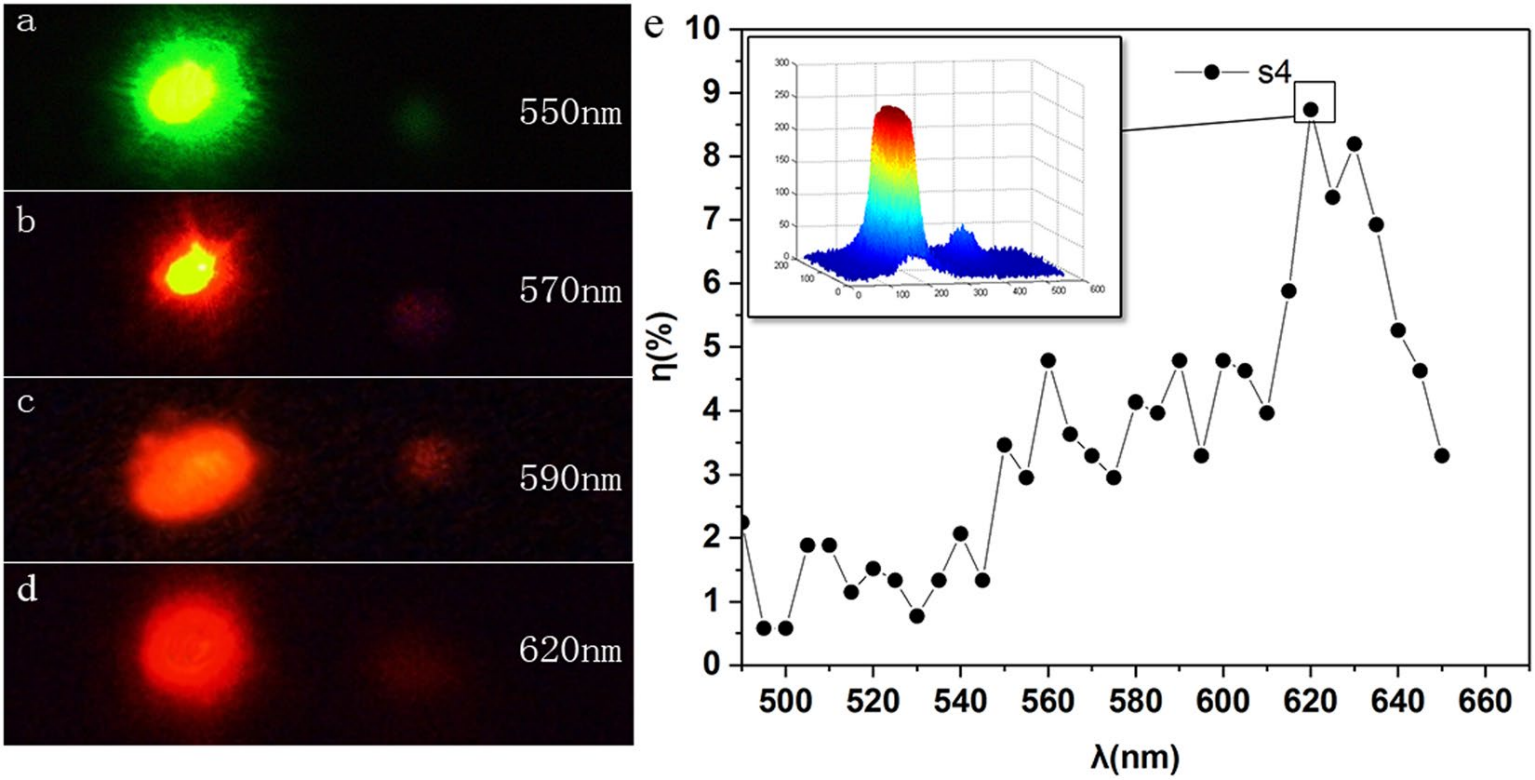
Experimental results of co- and cross-polarization transmission of (**a**) s1 at 550 nm, (**b**) s2 at 570 nm, (**c**) s3 at 590 nm, and (**d**) s4 at 620 nm (Visualization 1). (**e**) The conversion efficiency of s4 as a function of wavelength.

Transmitted spectral curve of the dendritic cell-cluster metasurface in Fig. 2 shows that in addition to normal co-polarized transmitted light, a cross-polarized transmitted light is obtained when wavelength of incident light is within resonant wavelength of the sample. When wavelength of incident light and resonant wavelength of samples are inconsistent, transmitted light is co-polarized along the original propagation path. Responses of the four samples are measured to illustrate the connection between resonant wavelength of dendritic cell-cluster metasurface and operating wavelength of cross-polarization conversion. Results are shown in Fig. 4. Cross-polarized transmitted light of sample s1, which operates at 550 nm wavelength, is obtained (Fig. 4a). Response wavelength of measured sample approximates 520 nm (Fig. 2b). Cross-polarized transmitted lights of samples s2, s3, and s4 are shown in Fig. 4b,c,d, respectively. Images of transmitted light spots are imported to MATLAB, and image-processing function of MATLAB is used. Intensities of co- and cross-polarized transmitted lights are obtained with 3D distribution figures (inset of Fig. 4e). Conversion efficiency is defined as the ratio of the power converted into anomalous component over the power of overall transmittance. Conversion efficiency of s4 is 8.7% at resonant wavelengths and 2% at non-resonant wavelengths. It is noted that Qin *et al*.^20^ proposed the periodical V-shaped metasurface operating in near-infrared provides about 36.5% conversion efficiency. Conversion efficiencies of s1, s2, and s3 equal 11.39%, 15.7%, and 17.9%, respectively. Obviously, the efficiency of s4 is the lowest, which means that its experimental phenomenon is the weakest. We chose the measurement of s4 to show that the measurement process is most universal. Figure 4e presents conversion efficiency as a function of wavelength. Cross-polarized transmitted light yields lower intensity than co-polarized light. Cross-polarized light intensity of sample s3 is the highest among all measured samples (Fig. 4c); sample s3 also presents the highest transmission coefficient (Fig. 2b). In summary, resonant wavelength of dendritic cell-cluster metasurface is consistent with operating wavelength of cross-polarization conversion using the dendritic cell-cluster metasurface. Response wavelength of the dendritic cell-cluster metasurface is controlled during preparation. Cross-polarized transmitted light operating at different wavelengths is obtained using the response of dendritic cell-cluster metasurface at the corresponding wavelength.

## Conclusions and Prospects

In this study, simulation and experimental results show that incident light is perpendicular to the dendritic metasurface. When wavelength of incident light coincides with the resonant band of the sample, cross-polarized transmitted light tilts out. Reference^24^ showed that metasurface should conform to generalized Snell’s law and present a phase jump in the corresponding band. The dendritic metasurface is an effective visible-light metasurface which been indicated in reference^36^. Thus, dendritic cell-cluster metasurface can realize tilted propagation of cross-polarized light in relation to the main polarized light. Refraction angle of the co-polarized transmitted light is 0°, whereas that of the cross-polarized transmitted light approximates 60°. A certain deviation exists between experimental and simulation results mainly because the dendritic cell-cluster metasurface sample prepared by electrochemical deposition does not completely agree with the model in simulation. Simulated and experimental results reveal that the dendritic cell-cluster metasurface achieves cross-polarization conversion of linearly polarized incident light in the resonant frequency of the sample. As expected, conversion efficiency of cross-polarization is enhanced by improved experimental design and preparation technique.

In conclusion, we proposed a dendritic cell-cluster metasurface with asymmetric quasi-periodic structure, and cross-polarization conversion is achieved at the visible light in transmission mode. Silver dendritic cell-cluster metasurface is prepared by electrochemical deposition based on the bottom-up concept. Numerical simulation and experiments confirm that when the wavelength of incident light coincides with sample resonant wavelength, co- and cross-polarization transmissions are obtained. When the linearly polarized incident light perpendicularly passes through the silver dendritic cell-cluster metasurface, co-polarization light is perpendicular to the interface, and a tilted cross-polarization is emitted. The highest conversion efficiencies up to 17.9% in 590 nm. Further improvements in preparation of dendritic cell-cluster metasurfaces may enhance conversion efficiency and increase potential applications of this novel metasurface in light manipulation.

### Appendix

In this appendix, we give a detailed description of the dendritic structure geometrical parameters in simulation (Fig. 1b). The three types of dendritic structure are three branches, four branches and five branches, which is composed of narrow and broad rods. The rod thickness is 12 nm. Grading outwardly from the center of the structure. The first type of dendritic structure which marked in red dashed box consists of three main branches, with three levels from the center point outward. The length of the rod Δ*l*_1_ = 105.97 nm. The width of the narrow and the broad rod is *w*_n_ = 13.5 nm, *w*_b_ = 26.9 nm. The angles between the branches in every level are same, which is ∠1 = 120°, ∠2 = 60° and ∠3 = 48°. The second type of dendritic structure which marked in green dashed box consists of four main branches, with two levels from the center point outward. The length of the rod Δ*l*_2_ = 122.7 nm. The width of the narrow and the broad rod is *w*_n_ = 15.6 nm, *w*_b_ = 31.1 nm. The angles between the branches in first level are ∠1 = 120°, ∠2 = 60° and ∠3 = ∠4 = 90°, and angles in second level is ∠5 = 36°. The third type of dendritic structure which marked in blue dashed box consists of five main branches, with two levels from the center point outward. The length of the rod Δ*l*_3_ = 120 nm. The width of the narrow and the broad rod is *w*_n_ = 15.2 nm, *w*_b_ = 30.5 nm. The angles between the branches in the first level are ∠1 = ∠2 = 90° and ∠3 = 58°, ∠4 = 72°, and angles in the second level is ∠6 = 36°.

## Electronic supplementary material

 Supplementary Video
